# Current challenges and future directions of ATMPs in regenerative medicine

**DOI:** 10.1016/j.reth.2025.06.017

**Published:** 2025-07-09

**Authors:** Fatemeh Abbasi Kakroodi, Elaheh Khodadoust, Marzieh Alizadeh, Reyhaneh Sadat Hayaei Tehrani, Pedram Asadi Sarabi, Mohammad Rahmanian, Massoud Vosough

**Affiliations:** a Department of Applied Cell Sciences, Faculty of Basic Sciences and Advanced Medical Technologies, Royan Institute, ACECR, Tehran, Iran; b R&D Department, Royan Stem Cell Technology Company, Tehran, Iran; c Department of Regenerative Medicine, Cell Science Research Center, Royan Institute for Stem Cell Biology and Technology, ACECR, Tehran, Iran; d Department of Stem Cells and Developmental Biology, Cell Science Research Center, Royan Institute for Stem Cell Biology and Technology, ACECR, Tehran, Iran; e Student Research Committee, School of Medicine, Shahid Beheshti University of Medical Sciences, Tehran, Iran; f Gastroenterology and Liver Diseases Research Center, Research Institute for Gastroenterology and Liver Diseases, Shahid Beheshti University of Medical Sciences, Tehran, Iran; g Experimental Cancer Medicine, Institution for Laboratory Medicine, Karolinska Institute, Stockholm, Sweden

**Keywords:** Translational medicine, Manufacturing challenges, Advanced therapy medicinal product, ATMPs challenges

## Abstract

Advanced therapy medicinal products (ATMPs) represent a groundbreaking category of medications that utilize biological-based products to treat or replace damaged organs. It offers potential solutions for complex diseases through gene therapy, somatic cell therapy, tissue engineering, and combined therapies. Although ATMPs have offered significant improvement for a variety of severe illnesses, their progressive development is faced with numerous challenges. Some of these challenges are current complexities in their manufacturing, such as scaling up, scaling out, product efficacy, packaging, storage, stability, and logistic concerns. Other challenges include manufacturing, efficacy, and scaling up of ATMPs, as well as establishing Good Manufacturing Practice (GMP)-compliant processes that align with product specifications derived from non-clinical studies conducted under Good Laboratory Practice (GLP). Additionally, safety concerns such as tumorigenesis are potential. Regulatory and ethical concerns, along with the need for standardization and clear clinical guidelines, are also critical obstacles. To address these challenges, novel technologies such as organoids, artificial intelligence, dynamic culture systems, and biobanking are being explored, providing new opportunities to enhance the consistency, scalability, and precision of ATMP production. Development in artificial intelligence (AI) technology helped scientists to address monitoring concerns, automation, and data management. Introducing advanced guidelines in biobanking helps researchers to overcome the storage and stability concerns. Organoid technology holds a significant promise in overcoming the challenges associated with preclinical and modeling of ATMPs by providing more accurate models for diseases, drug screening, and personalized medicine. This article reviews the current challenges in ATMP manufacturing and application, highlights the advancements in technology that are paving the way for improved therapeutic strategies, and offers future perspectives on overcoming these barriers.

## Highlights

• Challenges include manufacturing, efficacy, and scaling up of ATMPs.
• AI and organoid technologies are addressing ATMP production hurdles.
• Tumorigenesis and safety concerns remain significant in ATMPs.
• Regulatory guidelines and innovations shape the future of ATMPs.

## 1 Introduction

Development of regenerative medicine and emerging novel ATMPs shed light on current medical challenges in translational research [1,2]. Application of ATMPs represents a significant and innovative medical treatment category that utilizes biological materials specifically biomolecules, scaffolds, live cells, tissue replacements. This review adopts the European Union/United Kingdom regulatory framework for ATMPs, which includes Gene Therapy Medicinal Products (GTMPs), Somatic Cell Therapy Medicinal Products (sCTMPs), Tissue-Engineered Products (TEPs) and Combined ATMPs [3]. Regulatory classifications and terminology may differ in other regions, such as the US or Japan. These products are designed to treat and prevent diseases by targeting the root causes rather than simply relieving symptoms. However, several challenges arise during ATMP production. These challenges include the hurdle of Good Laboratory Practice (GLP to Good Manufacturing Practice (GMP) transition, manufacturing complexities, stringent regulations, cost-effectiveness, and various preclinical and clinical issues that hinder the clinical application of these products.

This is mainly attributed to the complicated nature of these products, which involves the properties of their cellular components and the legal implications associated with them as pharmaceutical products [4]. Ensuring the manufacturing process is validated to maintain consistent quality, safety, and effectiveness of products, which is a crucial part of the transition to GMP [5]. Since these products involve living organisms, their storage and transportation requirements are more complex than those of conventional pharmaceuticals. Maintaining optimal conditions throughout the supply chain is a significant challenge that must be addressed in regenerative medicine.

There is a growing need to establish more rigorous, precise, and standardized criteria for evaluating the quality of pharmaceutical products. This is particularly the same for ATMPs, where the current assessment of safety and effectiveness in the final formulations is not as robust as it could be. Regulatory bodies overseeing drug production, such as the Food and Drug Administration (FDA), are exploring new guidelines for innovative products. The uniqueness of these products may create difficulties in applying many of the current regulations that govern them.

By consistently implementing these requirements throughout the manufacturing process, we can ensure the functionality and integrity of the products. This consistently ensures we meet the specifications across different batches.

In this study, we aimed to comprehensively explore the current challenges associated with the development, manufacturing, regulation, and clinical application of ATMPs, while also highlighting emerging technologies and future directions that may help overcome these obstacles in the field of regenerative medicine. Notably, GTMPs for *in vivo* gene therapies (e.g., viral vector-based treatments) fall outside the scope of this discussion.

## 2 Current challenges

### 2.1 Challenges in translating non-clinical GLP results into GMP-compliant manufacturing processes

The successful translation of therapies in regenerative medicine from the laboratory to the clinic faces significant challenges. A critical barrier is implementing GMP-compliant manufacturing processes that reliably meet the quality specifications defined during product development, including data from GLP-compliant non-clinical studies [6]. GLP focuses on protecting scientific data from contamination and ensuring that the data generated is accurate and reliable. GLP is used in preclinical development, where nonclinical laboratory safety studies support the development of new pharmaceuticals and biotechnology products [7]. GMP protects the product from contamination and ensures it meets the required standards. GMP is primarily used in manufacturing facilities where products are produced for commercial sale [8]. First, securing a reliable supply of raw materials, reagents, and other critical components that meet GMP standards can be a complex logistical challenge, especially for novel or specialized products. Strategic partnerships and supply chain management strategies to secure reliable sources of GMP-compliant raw materials and components will be a solution to overcome this challenge [9]. Transforming cell culture for laboratory research into a product that can be used for medicine is associated with many challenges. Cells derived from patients or donors can exhibit significant variability, quality, potency, and stability. Ensuring reproducible manufacturing processes that can accommodate this variability is a considerable challenge. Standardized cell characterization and quality control assays could be helpful to ensure consistent cell product quality [10]. Robust quality control, such as in-process testing, real-time release criteria, and stability studies, is essential for ensuring the stability and reliability of the final product [9]. Scalable cell expansion is another critical challenge. Large cell volumes for clinical applications often require extensive cell expansion, which can change the cells' phenotype and functionality. Developing scalable, GMP-compliant cell expansion protocols, such as automated closed-system bioreactors, could solve this challenge [11]. Also, GMP-compliant facilities and equipment must be designed to reduce the risk of contamination and ensure product integrity. Adapting existing research infrastructure to meet these requirements can be a significant undertaking. Modular, flexible facility and equipment designs that can be easily adapted to meet GMP requirements [9,12]. Validating the manufacturing process to ensure consistent product quality, safety, and efficacy is critical in the transition to GMP. This requires extensive testing and documentation, which can be challenging. Comprehensive process validation protocols and quality management systems to ensure product consistency and reliability could be helpful [5].

Finally, while GLP governs the reliability of non-clinical safety/efficacy testing, GMP ensures the reproducible production of clinical-grade ATMPs. The manufacturing process must be designed to consistently achieve the product's critical quality attributes (CQAs), which are initially identified through GLP studies and further refined during chemistry, manufacturing, and control (CMC) development.

### 2.2 Manufacturing challenges

#### 2.2.1 Safety concerns

One of the most reported challenge of ATMP based on a survey in Europe has been manufacturing [13]. Regenerative medicine products must be free of any contamination. However, traditional sterilization methods such as filtration are not feasible due to the size of this product. Additionally, heat or radiation sterilization would compromise the viability of the cells. As a result, the manufacturing process for cell-based products must occur under aseptic conditions. To ensure the effectiveness of this aseptic processing, it is essential to validate the process through a simulation test, commonly referred to as a media fill [14]. This contamination includes anaerobic and aerobic bacteria, fungi, mycoplasma, and endotoxin. One solution to this challenge is to produce these products under controlled conditions. The people involved in the production process, raw materials, and all production stages must be controlled, and all production processes must be validated. Periodic environmental monitoring of the production site is another way to reduce contamination and ensure product safety. Closed and automatic systems can also be used to reduce the risk of contamination.

In regenerative medicine, one of the significant challenges is the risk of tumorigenesis, which refers to the potential transformation of stem cells into neoplastic (tumor) cells during therapy. This risk is a critical concern because the regenerative potential of stem cells can act as a double-edged sword, offering healing possibilities but also carrying the possibility of inducing tumor formation [15]. For pluripotent stem cell (PSC)-derived products, *in vivo* teratoma formation assay is used to validate pluripotency of PSCs as their starting materials and detect residual undifferentiated PSCs in the drug products [16]. For somatic cell-based therapies, tumorigenicity is assessed using *in vivo* studies in immunocompromised models (e.g., NOG/NSG mice) rather than teratoma tests. Regarding *in vitro* safety testing, conventional soft agar colony formation assays have limited sensitivity for detecting rare transformed cells in therapeutic products. More sensitive methods such as digital soft agar assays or cell proliferation characterization test are now recommended [[17], [18], [19], [20]].

The genetic instability of cells caused by successive cultures is one of the other challenges that can be overcome by performing tests such as cell karyotype and selecting genetically stable cells [21].

#### 2.2.2 Efficacy challenges

Proving the efficacy of regenerative medicine products is one of the most critical issues in translational medicine [22]. A major challenge in evaluating efficacy is proving long-term clinical benefit through well-structured clinical trials. This is particularly difficult for ATMPs, which often focus on rare diseases with limited patient populations, making it hard to gather statistically robust data [23,24]. The limited availability of clinical samples, along with complexities in designing trials and selecting appropriate endpoints, raises concerns about the reliability and durability of therapeutic outcomes [25]. Furthermore, difficulties in clearly defining and assessing the mechanism of action and potency pose additional obstacles to confirming clinical effectiveness [26,27].

#### 2.2.3 Scaling up concern

Scaling up the manufacturing of ATMPs is a multifaceted challenge involving technical, regulatory, and financial aspects. The most critical scale-up concern for ATMPs is demonstrating product comparability after manufacturing process changes. Regulatory authorities in the US, EU, and Japan have issued tailored guidance (FDA 2023, EMA 2019, and MHLW 2024, respectively) to address these challenges. These documents emphasize risk-based comparability assessments, extended analytical characterization, and staged testing to ensure changes which do not impact safety or efficacy. For example, the FDA recommends a tiered approach for reporting changes, while the EMA highlights the need to identify CQAs most susceptible to process variations. Harmonization remains limited, however, with regional differences in stability requirements and acceptance criteria for autologous therapies. Developers are advised to engage regulators earlier particularly for high-risk changes (e.g., scale-up from research-grade to GMP bioreactors), to avoid clinical holds or non-approval due to failed comparability [[28], [29], [30]]. One of the primary technical difficulties is shifting from small-scale production—often conducted in academic or clinical settings—to large-scale industrial processes, all while maintaining product consistency, safety, and efficacy. For example, Berisa et al. [31] showed that bioengineered corneal epithelium could be successfully produced in a mini cleanroom isolator under GMP conditions, which helped lower production costs. Nonetheless, expanding such systems introduces significant challenges, including sustaining sterile environments, ensuring consistent quality across production batches, and meeting the stringent quality control standards required for ATMPs. These issues are further intensified by the inherently laborious and costly nature of ATMP production, as noted by Haeusner et al. [32]. Economic considerations add another layer of complexity to scaling up ATMP production. Expanding manufacturing capacity carries substantial financial risk, especially given the exceptionally high treatment costs—sometimes reaching up to $2 million per patient [33]. Champion et al. [34] highlighted those analyses of clinical and cost-effectiveness, as well as evaluations of budget impact, are essential in determining the public health viability of scaling up. These economic assessments are pivotal, as they can shape pricing strategies and reimbursement frameworks, ultimately influencing the accessibility of these advanced therapies for patients. This scale-up process may result in variations in product quality, especially in the context of cell and gene therapies, where accurate dosing is essential [13]. Companies often face difficulties in maintaining sterility and ensuring comparability between production processes, especially for autologous therapies that are designed for specific patients [35].

#### 2.2.4 Storage condition

Because regenerative medical products are produced to be commercialized and enter the market, storing them for a certain period is necessary. Due to the sensitivity of these products, this storage requires special conditions, which is one of the existing challenges. Product cryopreservation is one of these challenges because this method is costly, unreliable, and requires bulky equipment. This method also induces significant stress on cells during freezing and thawing [36]. The cryopreservation media must be able to keep these products viable for a long time and cause the most minor damage to them. To protect cells from freezing stress and thawing, the freezing media contains agents called cryoprotective agents)CPA) [37]. Dimethyl sulfoxide (DMSO) and glycerol are the most common CPAs used. CPAs like DMSO can be toxic to cells, which is another challenge. For mesenchymal stromal cells (MSCs), numerous studies have indicated that cryopreservation may lead to diminished clonogenic potential and a delayed restoration of full therapeutic function after thawing. This can compromise the effective clinical dose and ultimately impact the overall efficacy of the ATMP [38]. One solution to these challenges is research and study to optimize the freezing media and validate the freezing methods according to guidelines. Also, the cell's exposure time to CPAs should be as short as possible.

#### 2.2.5 Packaging challenges

After determining the product production process, it is necessary to decide on the packaging of the final product. There are two types of packaging for the product: essential packaging, such as a bag, which is designed based on the administration route and dose, and secondary packaging, such as a box with a label [39]. Because these products are composed of living cells, it is very challenging to design and choose their packaging so that the viability and efficiency of the cells are not compromised. Additionally, these packages must be made of non-toxic and biocompatible material to ensure that they do not adversely affect the product.

#### 2.2.6 Stability concerns

The stability of regenerative medicinal products, particularly cell-based therapies, is crucial to their development and application. Maintaining the strength of these products involves several challenges and considerations**.** Determining the storage time, temperature, and final product formulation are challenging factors affecting this treatment's stability.

One way to solve this challenge is to perform product stability tests according to existing guidelines [39]. The stability test of the final product should be done to obtain the optimal temperature conditions for its storage and transportation so that its specification does not change [40]. Moreover, a stability test helps to determine the product expiration date during its storage and its shelf life at the time of administration to the patient [41].

#### 2.2.7 Logistic challenges

Cell therapy products require unique logistics features to maintain viability and ensure timely patient delivery. For example, the autologous treatment workflow includes a cryogenic logistics cycle that consists of the clinic or hospital where the patient will receive treatment and the manufacturer producing the therapy. This cycle starts with collecting a biopsy or sample from the patient, processing the product, and administering it to the patient for treatment.

On the other hand, due to the nature of regenerative medicine products that are living cells, transferring these products to the patient is complex and challenging compared to other process treatments.

This challenge exists for the distribution of both autologous and allogeneic products. However, the severity of this challenge is more significant in autologous products due to sampling from the patient and transferring the sample to the production site [42]. According to existing guidelines, validating the time and temperature of patient sample transfer and product transfer is a solution to overcome this challenge.

### 2.3 Preclinical challenges

The preclinical hurdles may receive less attention in the literature due to the flexible nature of this phase. Preclinical research is less structured and has less oversight, unlike the clinical phase, which is governed by strict protocols and subphases. While the experiments themselves follow rigorous scientific methods, the basic scientists have more flexibility in designing and conducting studies, making it difficult to recognize explicit challenges [43].

However, researchers have identified three primary risks linked to regenerative medicine technologies, including tumorigenicity, immunogenicity, and the risks associated with the implantation procedure [44]. Addressing these risks requires a comprehensive understanding of the underlying mechanisms of action [45].

The scientific community acknowledges the difficulties associated with identifying mechanisms of action; nevertheless, it is necessary to enhance efforts in this area, as such progress will inform decisions regarding translational applications and manufacturing processes. Consequently, it is vital for basic scientists involved in technology development to prioritize the clarification of these mechanisms. Additionally, a significant preclinical challenge is the absence of efficient translation processes for basic scientists. Although there has been a rise in the participation of basic scientists in the translational process and a more practical approach in recent years, there continues to be a widespread lack of incentives and resources to support the translation of their research [43].

Another preclinical challenge that has gained attention in recent years is the inadequacy of suitable preclinical testing models. It is frequently observed that innovative therapies perform well in laboratory settings but subsequently fail in more extensive animal studies or clinical trials. This issue is partly due to a limited understanding of mechanisms and a lack of appropriate *in vitro*, *in vivo,* and *ex vivo* models. Well-validated preclinical models enable us to accurately assess the efficacy of novel therapies and forecast their clinical success. Currently, we may be misjudging the potential of these therapies. The lack of appropriate models notably hinders the collection of dependable data concerning the mechanisms of action of regenerative medicine therapies, as there may be inconsistencies between preclinical and clinical settings [46].

### 2.4 Clinical challenges

Notably, rigorous and reliable preclinical data are necessary or utilizing ATMPs in clinical settings. However, the quality of evidence and reliability of preclinical studies are growing concerns [47]. A policy report published by the European Academies Science Advisory Council (EASAC) reported that most Cochrane reviews in regenerative medicine had indicated low levels of evidence and considerable risks of bias in the preclinical studies [48]. Publication bias and selective reporting are ethical issues that can mislead scientists, waste financial resources, and, more importantly, put patients’health at risk [49]. On the other hand, when it comes to clinical interventions, a potential challenge is deficiencies in determining the optimal and reliable outcomes for evaluating the efficacy of regenerative medicine products. Regulatory parties, peer-reviewed journals, and scientific societies should ensure transparent, well-designed, and well-conducted preclinical and clinical studies to avoid any harm to human subjects. The International Society for Stem Cell Research)ISSCR(has published a guideline recommending standardized biomedical research and its clinical translations [50]. Registers also facilitate monitoring clinical research protocols and avoid selective reporting [51]. Despite guidelines and regulatory agencies, regenerative medicine has a lot of uncertainty. For instance, in cell-based therapy, optimal cell dosage, frequency and interval of administration, and route of administration are not apparent in the clinical setting, and scientists must tackle this with substantial uncertainty while assuring the least harm. Efforts have been made to address uncertainties, such as using network meta-analysis to compare various findings [[52], [53], [54]]. However, the absence of clinical practice guidelines for regenerative therapies has remained unresolved.

Although preclinical research provides valuable information on a product's safety and efficacy, it faces substantial limitations in accurately replicating human body conditions [55]. For instance, studying the pharmacokinetics or biodistribution of systematically injected stem cells is not feasible in *in vitro* and preclinical studies [56].

In *ex vivo* gene therapy and cell therapy, maintaining the proliferative capacity and regenerative potential of the transplanted cell and preventing their senescence and apoptosis is a challenge in clinical studies [47]. Scientists should consider a therapeutic strategy that enhances the growth advantage of cell engraftment, like cell surface molecular engineering, cytokine pretreatment of cells, and improving drug delivery using nanoparticles [57]. One of the first practices in regenerative medicine is transplanting stem cells following irradiation-induced damage to the primary bone marrow cell that facilitates the constitution of engrafted cells [58]. Hepatocyte engraftment following local irradiation also enhances the initial homing and integration of transplanted cells into the liver tissue [59]. However, irradiation causes substantial tissue damage and does not apply to vital organs like the heart or brain.

The nature of a disease and the target tissue are essential factors in designing a therapeutic approach and clinical research protocols. Some studies encourage the application of regenerative medicine in more severe disorders due to ethical and financial limitations [47]. Individuals with the same eligibility criteria with a common disease receiving one particular therapy demonstrate various responses, which are theoretically explainable by personalized medicine. This issue causes a dilemma in regenerative medicine. Large-scale manufacturing costs less, and it would be accessible for more patients, while precision regenerative medicine is customized and more efficient but costs more and demands specific diagnoses.

Clinical studies must address ethical challenges related to patients’ rights and safety. Researchers should empower patients by providing precise and reliable information, enabling them to make well-informed decisions before giving consent [56].

Although the rationale and therapeutic effect of cell- and gene-based therapies are necessary for developing new regenerative therapies, the scale of these treatments is an important issue. The authorized products must be accessible reasonably unless it would cause ethical concerns about distributive injustice. This injustice can be caused by the high cost of these innovative products or the lack of needed technologies in most low- and middle-income regions.

### 2.5 Regulatory challenges

Due to the complexity of ATMPs and the strict requirements for their development and approval, the regulatory issues related to these products are complex and multifaceted. Key challenges include.

#### 2.5.1 Regulatory framework and compliance

Such products are subject to specific regulatory laws approved by the European Union (EU) and the United States of America (USA) to achieve the necessary standards for quality, safety, and efficacy. In the EU, Regulation (EC) No 1394/2007 governs ATMPs, requiring a centralized marketing authorization through the European Medicines Agency (EMA) [60]. This regulation necessitates comprehensive documentation and adherence to GMP, which can be particularly challenging for academic institutions and smaller companies lacking extensive regulatory experience [30].

#### 2.5.2 Documentation and expertise

A significant barrier is the requirement for detailed product-related documentation, such as the Investigational Medicinal Product Dossier (IMPD). Many academic centers often lack the expertise to prepare these documents adequately, making early interaction with regulatory bodies crucial for understanding requirements and receiving guidance [30]. The complexity of ATMP requires strong documentation that includes all the details of product quality and performance, which can be overwhelming for inexperienced developers.

#### 2.5.3 Clinical trial applications (CTA)

The preparation of CTAs is another critical hurdle. Regulatory authorities expect robust data on quality, non-clinical safety, clinical efficacy, and risk-benefit assessments before approving clinical trials. This expectation can be daunting for smaller developers who might lack the resources or expertise to gather such extensive data sets [30].

#### 2.5.4 Variability in regulatory standards

Differences in regulatory standards between regions, especially those between International Council for Harmonization of Technical Requirements for Pharmaceuticals for Human Use (ICH) founder countries/regions (e.g., EU vs. US) can also pose challenges for developers aiming for international markets. Understanding these nuances is essential for successful product development and commercialization [60]. Since numerous ATMPs are designed to treat rare diseases or conditions with significant unmet medical needs, it is crucial for regulatory authorities to revise and adapt their frameworks to address the distinct challenges and complexities these therapies present [23].

#### 2.5.5 Governance

**Fig. 1:**
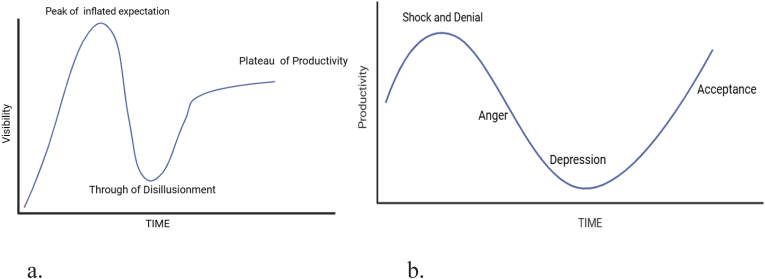
**a**. The Gartner Hype Cycle. **b.** The Kübler-Ross Change Curve.

Identifying and addressing challenges in regenerative medicine is impossible without a systematic approach and strong leadership. Regenerative medicine sparked a wave of excitement due to its therapeutic potential, leading to significant investment and high hopes in the field. According to Gartner's hype-hope cycle (Fig. 1a), emerging technologies often result in speculative investments and unrealistic expectations. After the initial disillusionment, the “hope” phase begins, where the true potential of the technology is understood and realistic, leading to sustainable development and adoption [61]. This curve mirrors the Kübler-Ross Change Curve (Fig. 1b), which reflects how communities experience new phenomena. Interpreting these patterns suggests that effective change management is essential for achieving a paradigm shift and embracing the change realistically and productively in regenerative medicine.

Effective change management requires active stakeholder involvement to support the transition process. It is crucial to identify different actors and stakeholders, promote effective communication with them, and understand their unmet needs and interests.

Medical practitioners and community members are the end-users of ATMP, whereas educating these populations is neglected. It's been suggested to integrate regenerative medicine into the curricula for medical education and professional training [51]. Training specialized workforces in regenerative medicine is a strategic investment. The university and industry collaboration faces other challenges, such as a lack of knowledge about product manufacturing and GMP and insufficient business expertise among academia [62]. To fill the gap between academia and industry and also translate research findings in clinical settings, medical education curricula should reflect new advancements in cell- and gene-based therapy in addressing severe and intractable diseases [63]. Additionally, universities with robust research infrastructures can collaborate with industrial entities due to their capacity to transfer knowledge [64].

The commissioner of the FDA in the United States, one of the founder countries/regions of ICH, reported insufficient oversight of unscrupulous private clinics offering unapproved regenerative treatments [65]. These illegal practices harm the patients' safety and undermine the public trust in regenerative medicine [49]. Legal enforcement by regional authorities is essential to stop these harmful and opportunistic actions. Raising community awareness is necessary to prevent malpractices, promote public engagement, and manage unrealistic expectations about these treatments [48]. As a critical stakeholder, the media plays a substantial role in enhancing patients’ awareness and fostering realistic, evidence-based expectations about the current potential of regenerative medicine [47]. Some studies recommend the academic centers implement strategies for monitoring press releases for fairness and accuracy (Table 1) [56].

**Table 1:** Summary of key challenges and strategic solutions in the development and application of ATMPs.

Challenge Area	Key issues	Suggested solutions
GLP to GMP transition	Difficulty in sourcing GMP-grade materials, variability in donor-derived cells, need for extensive validation and documentation	Develop scalable, closed-system bioreactors; establish strong QA/QC frameworks and modular cleanrooms
Manufacturing	Lack of feasible sterilization methods for living cells, risk of microbial contamination during open handling processes	Use automated, closed manufacturing platforms; routine environmental monitoring and aseptic process validation
Safety	Risk of stem cell tumorigenesis, accumulation of genetic mutations during *in vitro* expansion	For PSC products: Teratoma assays to detect residual pluripotent cells For somatic cell products: • In vivo tumorigenicity studies (NOG/NSG mice) • Digital soft agar or proliferation assays for transformed cells • Routine karyotypic analysis
Efficacy	Lack of standardized potency assays and predictive markers for therapeutic outcomes	Implement *in vitro* potency assays tied to mechanism of action; early-phase human trials for functional validation
Scaling up	Difficulty ensuring batch-to-batch consistency, maintaining product integrity in personalized (autologous) therapies	Use platform manufacturing approaches; invest in tech transfer protocols and real-time comparability analytics
Storage conditions	Cell damage during freezing/thawing, DMSO toxicity, high cost and complexity of cryopreservation infrastructure	Design optimized cryoprotective agents and short exposure protocols; adopt new cryopreservation techniques
Packaging	Designing biocompatible, sterile, and user-friendly primary and secondary packaging that preserves cell viability	Use non-toxic, tamper-evident packaging materials validated for storage conditions and dosing accuracy
Stability	Uncertainty in optimal storage temperatures and durations; degradation during transport	Conduct ICH-compliant stability studies under varied temperature and humidity; validate shelf life and transport conditions
Logistics	Maintaining viability during patient sample transport; validating cryogenic handling for both autologous and allogeneic products	Integrate digital tracking and validated logistics solutions for cold chain and just-in-time manufacturing
Preclinical	Insufficient models that mimic human physiology; poor predictability of therapeutic efficacy and safety	Advance 3D culture, organoids, and animal models with better translational fidelity
Clinical	Low-quality preclinical data, selective reporting, lack of dose optimization and trial standardization	Use registry-based monitoring; adhere to ISSCR and international clinical trial standards; meta-analysis for protocol harmonization
Regulatory	Complex CTA preparation, variability in global regulatory requirements, lack of in-house expertise in academic settings	Early collaboration with regulators; capacity-building programs in academic centers; international regulatory alignment
Governance	Lack of public and clinician awareness, insufficient ethical oversight, risk of hype and misinformation	Develop stakeholder engagement plans, training curricula, and ethical monitoring frameworks; regulate media narratives

**Abbreviations: ATMPs** (Advanced Therapy Medicinal Products), **GMP** (Good Manufacturing Practice), **GLP** (Good Laboratory Practice), **QA/QC** (Quality Assurance/Quality Control), **CPA** (Cryoprotective Agent), **DMSO** (Dimethyl Sulfoxide), **ICH** (International Council for Harmonization of Technical Requirements for Pharmaceuticals for Human Use), **ISSCR** (International Society for Stem Cell Research), **CTA** (Clinical Trial Application), **3D** (Three-Dimensional), **in vitro** (Outside a living organism), **in vivo** (Inside a living organism), **ex vivo** (Outside the organism but using living cells or tissues).

**Fig. 2:**
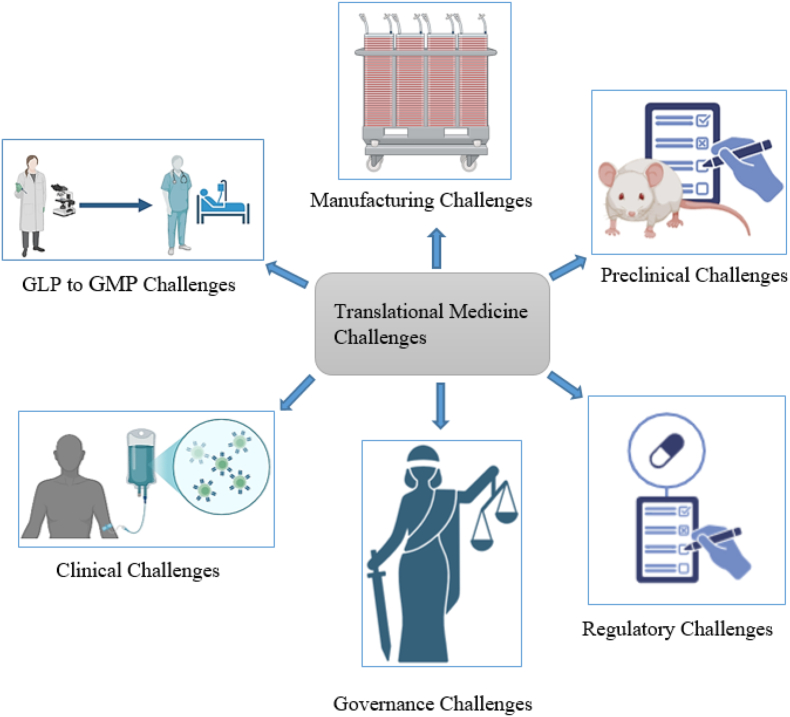
Some challenges of translational medicine.

Ethical concerns regarding regenerative research and therapy are significant challenges and responsibilities of policymakers, researchers, and practitioners, and governance has the critical duty of surveillance. Regulators must monitor and handle the probable conflict of interest in the competitive environment of regenerative medicine [66]. Currently, the results are heterogeneous across different clinical trials, but the results of efficacy and long-term benefits of regenerative therapies for incurable diseases mostly showed modest or no impact. On the other hand, the cost of regenerative studies and regenerative therapies is high. This issue raises an ethical concern about devoting a significant amount of budget to this field when it could have been invested in other promising efforts [47]. The limited funding requires well-informed policy-making based on the harm-benefit of an intervention. It is a potential challenge to keep the balance in investigating this novel technology and other fields of research while the appeal of regenerative medicine should not distract the policymakers from prioritizing the public good while still encouraging the pursuit of cutting-edge research and addressing complex questions (Fig. 2) [47].

## 3 Novel technologies to overcome current challenges

Novel technologies are increasingly being developed and implemented to address various challenges across multiple fields. The following section mentions ways to solve the challenges in the manufacture and quality characterization of ATMPs.

### 3.1 Organoids as development tools for ATMPs

Organoids serve as powerful tools to support the development and testing of cell therapy products, rather than as direct therapeutic agents. Their ability to mimic human physiology makes them invaluable for preclinical modeling, drug screening, personalized, and precision medicine approaches [67]. A notable advancement in regenerative medicine is the development of organoids. Organoids are three-dimensional structures composed of organ-specific cells that are derived from stem cells [68]. These cells are cultivated within a biological or synthetic scaffold that simulates the extracellular matrix (ECM), supplying essential proteins and growth factors required for cellular growth and differentiation [[69], [70], [71]]. In contrast to earlier approaches in regenerative medicine, such as cell transplantation and two-dimensional cell cultures, organoids maintain their functional characteristics and properties over extended periods. They exhibit significant genetic stability, as well as the capacity for expansion, differentiation, and self- organization [72].

Organoids can replicate various pathophysiological and structural attributes of human tissues and organs, and they are currently utilized as models for studying diseases. They can be derived from pluripotent stem cells (PSCs), as well as ESCs and adult tissue stem cells (ASCs) [73,74]. Remarkably, organoids can generate diverse organ models, ranging from the stomach and intestines to the brain [74,75]. Notably, these cells have the potential to develop into brain components in an *in vitro* setting, a process that occurs independently of the natural evolutionary cycle [74,76].

The newly introduced concept of “precision medicine,” also called personalized medicine, leverages advanced technologies such as DNA sequencing to highlight inter-individual differences as critical factors in the assessment and treatment of diseases. Precision medicine concentrates on DNA sequencing and genomic data as primary contributors to disease presentation and management variability. However, contemporary precision medicine encompasses a broader spectrum of human characteristics, including lifestyle choices, socioeconomic factors, family history, and genetic influences concerning diseases [[77], [78], [79]].

Traditional treatment methods have typically involved administering the same therapy to patients diagnosed with a specific type of disease. However, recent findings indicate that while particular therapies may be effective for some individuals, they do not yield the anticipated outcomes for others. This discrepancy can be attributed to individual variations, highlighting the necessity for personalized medicine, which advocates for a tailored approach of “one patient, one treatment” [80]. In this context, organoids emerge as promising candidates, as they can replicate the *in vivo* environment [81]. Particularly in oncology, it is essential to identify the most effective therapeutic strategy for each patient, ensuring maximum benefit with minimal risk. Despite identifying genetic alterations, many patients do not respond to treatments as anticipated. To tackle this issue, organoids derived from patient tumor samples, referred to as patient-derived organoids (PDO), have been developed [82]. These PDOs represent the primary tumors in an *ex vivo* setting, accurately mimicking the phenotypic and genotypic characteristics of the original tumors [70,83]. A PDO from an individual patient can function as a cancer biobank, supporting various objectives of precision medicine. Within this biobank, genetic materials are analyzed, drug sensitivity is assessed, and extensive drug screening tests are conducted [83,84]. Furthermore, the responses of cancer to different therapeutic approaches, including chemotherapy, radiotherapy, and combination therapies, are evaluated. Consequently, the most effective treatment for the patient is determined, and clinical trials have demonstrated that the outcomes of the selected therapy align with those predicted by the PDOs [70]. To sum up, while organoids are not yet standardized as clinical-grade therapeutics, their role in risk assessment of ATMP development is transformative. Future efforts should focus on improving organoid technologies’ reproducibility, scalability, and integration with regulatory frameworks to bridge the gap between preclinical and clinical studies.

### 3.2 Artificial intelligence

In recent years, the incorporation of AI technology into stem cell therapy, regenerative medicine, and drug development has advanced considerably. AI encompasses the creation of computer systems capable of executing tasks that typically necessitate human intelligence [85]. It has emerged as an essential component in conducting computational simulations and in silico studies within medical applications, providing numerous benefits, including reduced costs and expedited results when compared to traditional medical research methods, such as clinical and laboratory investigations [[86], [87], [88]]. Presently, various initiatives are underway to integrate AI across a broad spectrum of sectors, including but not limited to medicine, pharmaceuticals, and healthcare [89,90]. Within regenerative medicine, AI technologies enable the real-time observation of manufacturing processes, which is essential for ensuring product quality and uniformity. By examining extensive datasets produced during manufacturing, AI can forecast quality characteristics and facilitate automated modifications using feedback control systems. This feature allows for rapid detection of any deviations from optimal conditions. As a result, it reduces the extent of these deviations and enhances confidence in ATMPs [8].

The integration of AI allows for the automation of various manufacturing tasks, reducing human-induced variability and errors. Automated systems can manage complex production environments more efficiently than manual processes, leading to higher scalability and reduced operational costs. This is particularly beneficial in the context of personalized medicine, where production must adapt to unique patient-specific requirements [30].

AI can enhance and streamline processes such as the analysis of extensive datasets comprising molecular and genetic information, enabling the identification of patterns and correlations that may elude human researchers. Creating regenerative therapies requires examining intricate and voluminous data, a domain where AI technology can be effectively utilized. Notable applications of AI in regenerative medicine encompass disease modelling, drug discovery, tissue engineering, cell therapy, and personalized medicine [91].

Cell-based therapy represents a promising avenue in regenerative medicine, employing stem cells to repair damaged tissues and organs [91]. Stem cells are considered optimal candidates for restoring injured tissues and organs. Nonetheless, challenges persist, including the time-consuming and costly process of cultivating stem cells stably and the difficulties associated with inducing specific cell differentiation. Furthermore, despite ongoing clinical trials, a fully realized cure through stem cell therapy remains elusive [92]. A growing number of researchers are utilizing artificial intelligence to examine large datasets and facilitate the identification of the most suitable cells for individual patients. A primary benefit of integrating AI into stem cell therapy lies in its ability to forecast the most effective cell types by scrutinizing patients' genetic data and medical histories. This approach not only supports the identification of conditions necessary for the differentiation of specific cells and the optimization of cell cultures but also enables predictions of cell types based solely on their morphology [93]. In drug discovery, the focus is on identifying active compounds that demonstrate therapeutic efficacy for particular diseases. An increasing body of research has substantiated the role of AI in critical domains such as de novo drug design, target structure prediction, and the prediction of drug-target interactions [93].

In personalized medicine, accurately forecasting a patient's response to a specific treatment poses a considerable challenge due to the complexity of biological systems [94]. AI has the potential to address this issue by analyzing patient data to uncover patterns and correlations that can inform treatment outcomes. One method through which AI can contribute to personalized medicine is by evaluating a patient's genomic information. AI algorithms can detect genetic variations associated with specific diseases or treatment responses, thereby facilitating the creation of tailored treatment strategies based on the individual's genetic profile [93]. Recent advancements in omics technologies, including genomics, transcriptomics, epigenomics, proteomics, lipidomics, and metabolomics, offer the potential for detailed molecular profiling of complex diseases. Novel technologies like AI paved the way in classifying a disease into biomarker-guided clusters (40).

### 3.3 Biobanking in regenerative medicine

Regenerative medicine and tissue engineering primarily rely on using stem cells for patient treatment; however, they may also incorporate mature cells that are not typically classified as stem cells. Stem cells can be sourced from various body parts, including bone marrow, cord blood, and adipose tissue. While stem cells are frequently utilized in therapies immediately after isolation, there are instances where stem and progenitor cells are collected, processed, and cryopreserved for future use. Biobanking is a practical alternative to immediate therapeutic application, facilitating patient recovery, allowing time to determine optimal treatment strategies, and enabling multiple interventions without imposing additional inconvenience or risk on the patient.

Although routine bone marrow banking is uncommon, the practice of cord blood banking for transplantation and regenerative medicine has paved the way for establishing cord tissue banking for future applications in these fields. This banking process involves freezing samples, which can be preserved indefinitely, in contrast to the cold storage methods used for red blood cells. For adults without access to cord blood collected at birth, adipose tissue banking has recently emerged as a promising option, given its abundance of MSCs. Notably, frozen adipose tissue has been successfully thawed after being stored for up to three years and has been used to treat over 200 patients [95].

The motivations behind the establishment of a biobank are aligned with the evolving objectives of contemporary medicine and interdisciplinary practices, including personalized medicine, early detection of specific conditions such as cancer or genetic disorders, and predicting responses to targeted therapies based on pharmacogenetic principles [96,97]. One of the best initiatives is the “All of Us” research program in the USA, aiming to collect broad data such as lifestyle, environmental variables, biological characteristics, and health records from one million people with diversity to build a foundation for precision medicine (41). Biobanking is also an infrastructure for collecting and analyzing big data, which helps us develop personalized treatments (42).

### 3.4 Dynamic culture

**Fig. 3:**
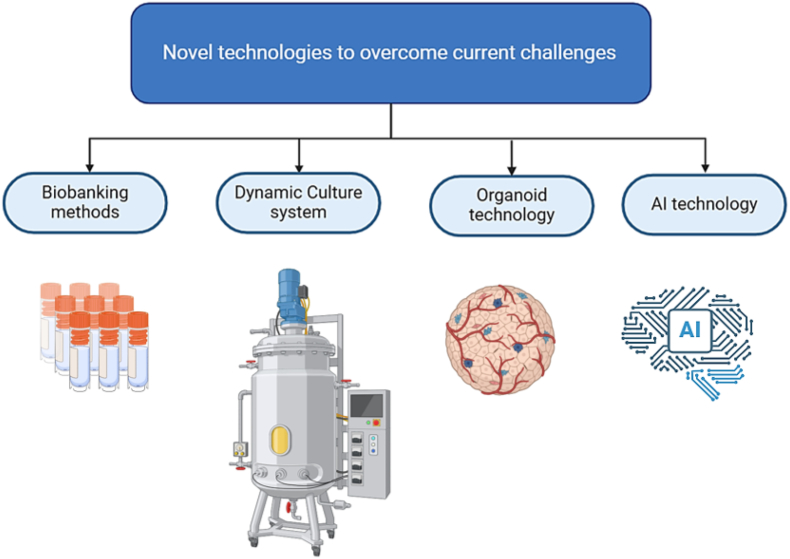
Novel technologies are increasingly being developed and implemented to address various challenges across multiple fields.

In order to be utilized effectively in regenerative medicine, primary MSC must be expanded in culture to adequate quantities. Traditional cell culture practices require frequent subculturing, as the growth of MSCs in size-restricted culture vessels is hindered by contact inhibition. The application of detrimental enzymes during passaging and the mechanical characteristics of standard culture vessels can alter the MSC phenotype. In contrast, dynamic culture expansion of MSCs minimizes the need for enzymatic passaging and results in greater MSC yields compared to conventional methods [98]. Dynamic culture systems allow for greater precision in managing cell growth conditions, resulting in enhanced final product consistency and quality. These systems can optimize cell growth and increase product yield through real-time monitoring and feedback mechanisms, effectively tackling challenges associated with scaling up production (Fig. 3).

## 4 Challenges of novel technologies

However, these novel technologies also have challenges. For example, the following can be mentioned.

### 4.1 Dynamic cultivation concerns

i. Mechanical and shear stress. High shear forces have the potential to cause damage or mortality in cells, especially in sensitive types such as stem cells. Although dynamic conditions can enhance mass transfer and nutrient delivery, it is essential to regulate these conditions carefully to prevent adverse impacts on cell viability [99].
ii. Optimization of Culture Conditions. Dynamic culture systems necessitate careful optimization of multiple parameters, such as flow rates, scaffold materials, and media composition. The interplay among these elements can significantly affect cell behavior, proliferation rates, and differentiation results. For example, distinct bioreactor types may produce different outcomes regarding cell growth and functionality, contingent upon their unique designs and operational conditions [100].
iii. Lack of Standardization. The absence of standardized protocols for dynamic cell culture practices is currently evident. This inconsistency may result in different outcomes across various studies, making it challenging to compare results and impeding advancements in the field. The development of universally recognized guidelines will be essential for promoting research and clinical applications related to dynamic cultures [101].
iv. Complexity in Scaling Up. The expansion of dynamic culture systems for large-scale production introduces further complexities. The shift from laboratory-scale to industrial-scale operations frequently uncovers constraints in existing bioreactor designs, including challenges in sustaining consistent conditions across the entire culture volume. Additionally, the labor-intensive requirements for monitoring and managing these systems may impede the implementation of cost-effective production methods [99].

### 4.2 Bio banking challenges

Also, the use of biobanks also has challenges, for example, presence of diverse data types, the potential for data loss, varying standards for data collection, and numerous ethical and legal considerations [102].

### 4.3 Organoid challenges

There are also challenges related to the use of organoids in translational medicine, including the diversity in their growth patterns, morphology, and functional attributes. This variability can be attributed to their self-organizing nature [103]. From the rest of the challenges, we can mention size limitation and limited life span, structural limitations such as lack of complexity and undefined matrices [104].

### 4.4 AI challenges

Despite the benefits of artificial intelligence integration into regenerative medicine, this integration also poses various challenges that must be tackled to guarantee its effective and ethical application. Among these challenges, the following can be mentioned.

i. **Data Quality and Volume**: AI systems require substantial amounts of high-quality data to operate efficiently. Insufficient data can result in inaccurate predictions and models. In the field of regenerative medicine, characterized by significant patient variability, obtaining well-organized data of sufficient quality remains a challenge [105].
ii. **Cellular Heterogeneity:** In stem cell therapies, cellular heterogeneity presents a considerable challenge. The variability in the properties of stem cells can influence treatment results, thereby complicating the ability of AI models to accurately forecast their efficacy. This inconsistency can be due to cellular senescence, which may hinder their therapeutic applications [106]**.**
iii. **Ethical and Safety Concerns:** The application of artificial intelligence in regenerative medicine presents ethical challenges, notably in relation to data privacy and the risk of bias inherent in AI algorithms. Additionally, there are safety concerns associated with the utilization of induced pluripotent stem cells (iPSCs), particularly with respect to insertional mutagenesis resulting from viral delivery mechanisms [107]**.**
iv. **Need for Standardization:** The absence of standardized protocols for the application of AI in regenerative medicine may result in varying outcomes among different studies and applications. It is crucial to develop clear guidelines to promote progress in this area [107]. Table 2 indicates the advantages and disadvantages of novel technologies in overcoming the translational medicine challenges**.**

**Table 2:** Advantages and disadvantages of novel technologies to support cell-based therapy products’ development.

Novel technologies	Advantages	Disadvantages
Organoids	✓ Mimicking the *in vivo* microenvironment ✓ As a cancer biobank and primary tumors in an *ex vivo* setting ✓ Alternatives to traditional models	✓ Growth patterns diversity ✓ Morphology diversity ✓ Functional attributes diversity ✓ Size limitation ✓ Limited life span ✓ Structural limitation
Artificial intelligence	✓ Ability of real-time observation of manufacturing processes ✓ Ability of forecasting the quality characteristics ✓ Facilitating automated modifications ✓ Reducing the extent of deviations and enhances confidence in ATMPs ✓ Facilitating the creation of tailored treatment strategies based on the individual's genetic profile	✓ Requiring a large volume and high-quality of high-quality data ✓ Ethical and safety concerns ✓ Lack of standardization for AI in regenerative medicine
Bio banking	✓ Helping to personalized treatments ✓ Predicting responses to targeted therapies based on pharmacogenetic principles ✓ Early detection of specific conditions such as cancer or genetic disorders,	✓ Presence of diverse data types ✓ the potential for data loss ✓ Varying standards for data collection ✓ Ethical and legal considerations
Dynamic culture	✓ Greater precision in managing cell growth conditions ✓ Optimize cell growth and increase product yield ✓ Real-time monitoring and feedback mechanisms ✓ tackling challenges associated with scaling up production	✓ Mechanical and share stress ✓ Interplay among dynamic culture elements ✓ Lack of standardization ✓Complexity in Scaling Up

**Abbreviations: AI** (Artificial Intelligence), **ATMP** (Advanced Therapy Medicinal Products), **in vivo** (Within a living organism), **ex vivo** (Outside the organism but involving living tissues or cells).

## 5 Conclusion and future perspectives

ATMP production and application may seem complicated, but their primary goal is simply enhancing people's health. But the obstacles encountered along the way have rendered the application of this treatment method challenging. Overcoming these challenges necessitate cooperation among various collaborators, such as regulators, industry participants, and funding organizations, to establish a conducive environment for effectively advancing these groundbreaking therapies. Also, the manufacturing of ATMPs is a highly regulated field that requires adherence to strict GMP guidelines and involves intricate processes aimed at ensuring product safety and efficacy. Additionally, ongoing advancements in regulatory policies and technological innovations continually shape this dynamic industry. Although these products promise the treatment of complex diseases, their development faces numerous regulatory hurdles that require meticulous planning, substantial investment, and collaboration with regulatory bodies.

On the other hand. Many cutting-edge therapies often show promising results in laboratory experiments but tend to fail during more extensive animal studies or clinical trials. This problem partly stems from insufficient mechanistic insights and a limited availability of suitable *in vitro*, *in vivo*, and *ex vivo* models. On the other hand, in developing these products in clinical studies, challenges such as uncertainty in the optimal cell dose, frequency and intervals of administration, and the route of administration in the clinical environment and uncertainty in the quality of preclinical studies can be mentioned. In conclusion, we need to remember that the challenges regarding regenerative medicine are complex problems, and they cannot be addressed simply without a deep understanding of the various challenges of the system and their interactions.

However, today, new technologies, such as artificial intelligence, the formation of biobanks, the development of organoids, and the cultivation of dynamic systems, can help overcome these problems and challenges. Developing vital manufacturing facilities that incorporate cutting-edge technologies, including artificial intelligence for process oversight and regulation, can significantly improve production efficiency and product quality [108]. Undoubtedly, these technologies present certain challenges; however, these obstacles can be addressed through advancements in the technologies themselves.

As this field progresses, clear regulatory guidelines for AI applications will be essential to maximize its advantages in the development of ATMPs.

## Authors’ contributions

The idea presented was conceived by M.V., while F.A. authored the manuscript with assistance from E.K., M.A. and R.H. M.R. and P.A. revised the article with respect to its composition and finally M.V. confirmed the composition of the article from a scientific perspective. M.V. and M.R. supervised the project.

## Declaration of Generative AI and AI-assisted technologies in the writing process

The authors employed QuillBot and ChatGPT for the final language editing of this paper. Following the use of these tools, they carefully reviewed and revised the content to ensure it met the required standards and take full responsibility for the published work.

## Funding

Not applicable.

## Declaration of competing interest

None.
